# Advances in Lycopene Production: From Natural Sources to Microbial Synthesis Using *Yarrowia lipolytica*

**DOI:** 10.3390/molecules30214321

**Published:** 2025-11-06

**Authors:** Paweł Moroz, Aleksandra Bartusiak, Julia Niewiadomska, Kacper Szymański, Tomasz Janek, Anna Kancelista, Anna Gliszczyńska, Zbigniew Lazar

**Affiliations:** 1 Department of Biotechnology and Food Microbiology, Wroclaw University of Environmental and Life Sciences, Chelmonskiego 37, 51-630 Wroclaw, Poland; pawel.moroz@upwr.edu.pl (P.M.); aleksandra.bartusiak@upwr.edu.pl (A.B.); 121174@student.upwr.edu.pl (J.N.); kacper.szymanski@upwr.edu.pl (K.S.); tomasz.janek@upwr.edu.pl (T.J.); anna.kancelista@upwr.edu.pl (A.K.); 2 Department of Food Chemistry and Biocatalysis, Wroclaw University of Environmental and Life Sciences, Norwida 25, 50-375 Wroclaw, Poland; anna.gliszczynska@upwr.edu.pl

**Keywords:** *Yarrowia lipolytica*, lycopene, short-chain fatty acids, phospholipids

## Abstract

Lycopene, a natural carotenoid with antioxidant and health-promoting properties, has attracted attention as a valuable compound for the food, pharmaceutical, and cosmetic industries. Conventional production methods based on plant extraction or chemical synthesis are limited by low yields, high costs, and environmental concerns. In this study, the oleaginous yeast *Yarrowia lipolytica* was engineered as an alternative microbial cell factory for sustainable lycopene biosynthesis using short-chain fatty acids (SCFAs)—such as acetate, butyrate, and propionate—as inexpensive, renewable carbon sources. Four heterologous genes from *Pantoea agglomerans* (*crtI*, *crtB*, *crtE*, and *idi*) were codon-optimized and integrated into the *Y. lipolytica* genome using different expression systems, including the Golden Gate Assembly strategy. Among the tested strains, PS05/4lyc/GGA, characterized by enhanced phospholipid biosynthesis, demonstrated the highest lycopene yield of 462.9 mg/g dry cell weight and a titer of 3.41 g/L on butyrate medium—values comparable to or exceeding those reported for bioreactor-scale fermentations. The results indicate that co-activation of phospholipid and carotenoid biosynthesis pathways creates favorable intracellular conditions for hydrophobic pigment accumulation. Moreover, the use of SCFAs improved acetyl-CoA availability and redirected carbon flux through the mevalonate pathway, enhancing productivity. Strains with elevated membrane lipid biosynthesis also exhibited higher metabolic stability and stress tolerance.

## 1. Introduction

Lycopene, a naturally occurring carotenoid pigment, has attracted considerable attention due to its strong antioxidant properties and numerous health benefits [1]. It is found mainly in tomatoes, red fruits and vegetables [2], but its production under natural conditions can be challenging [3].

Lycopene is a polyunsaturated hydrocarbon made of 40 carbon atoms and 13 double bonds, 11 of them conjugated. This structure gives lycopene its red color and strong antioxidant activity [4,5]. The presence of conjugated double bonds makes lycopene one of the most efficient naturally occurring antioxidants in food [5].

Lycopene is known for its positive effects on human health [6,7]. It exhibits strong antioxidant activity—combating oxidative stress, inhibiting low-density lipoprotein (LDL) oxidation, and preventing cardiac remodeling [6]. As a result, lycopene has been studied as a preventive and therapeutic agent for cardiovascular diseases, certain cancers, and neurological disorders [8,9]. Carotenoids such as lycopene can embed within phospholipid bilayers, where they stabilize membrane structure and improve its fluidity. Their presence also limits oxygen diffusion into the hydrophobic core of the membrane [10,11]. In this way, carotenoids support the protection of biomembranes against oxidative stress and can improve their function in living cells. The antiproliferative and anti-inflammatory properties of lycopene form the basis of its use in studies on cancers such as prostate, lung, and breast cancer [12]. Research indicates that lycopene can induce apoptosis in cancer cells, limit metastasis, and reduce levels of inflammatory cytokines [12]. Moreover, its photoprotective activity contributes to maintaining skin health and may protect against UV-induced damage [5]. Regular lycopene intake ranging from 2 to 20 mg per day has been associated with a reduced risk of these diseases and may support their treatment [8].

The growing interest in natural food additives has driven the search for efficient and environmentally friendly methods of lycopene production [13]. Due to its high content in plants, the primary industrial method of lycopene production remains extraction from plant biomass. Tomatoes are the most commonly used raw material, as they are rich in lycopene, relatively inexpensive, and readily available. However, the extraction process is associated with several limitations. The efficiency of lycopene recovery strongly depends on environmental factors, leading to variability in raw material quality. Moreover, this method involves high consumption of organic solvents, low process specificity, and potential environmental harm [14].

As an alternative to plant extraction, chemical synthesis offers independence from seasonal variations and raw material supply. However, the process remains inefficient and costly, which limits its industrial use [15]. In response to these challenges, microbial production of lycopene has gained increasing attention as a more sustainable and economically viable approach. Various microorganisms capable of synthesizing carotenoids, including algae (*Chlorella*, *Dunaliella*) and yeasts (*Rhodotorula*), are used in biotechnological processes [16]. Filamentous fungi such as *Blakeslea trispora* have also been employed in industrial biotechnological production due to their high carotenoid yields, including lycopene [14].

Recent advances in genetic engineering have significantly contributed to the development of biotechnological methods for lycopene production. Research focuses on constructing microbial strains capable of efficient carotenoid biosynthesis, thereby reducing dependence on plant sources and improving industrial productivity. In this context, particular attention has been given to GRAS (Generally Recognized As Safe) organisms, which are considered safe for use in the food and pharmaceutical industries [17,18].

Among the microorganisms used in biotechnology, the yeast *Yarrowia lipolytica* holds a particularly important position. It has emerged as one of the most promising production platforms for lycopene and other high-value biomolecules [19]. This species shows high metabolic flexibility and can utilize a wide range of carbon sources, including industrial waste substrates. These traits support the development of low-cost and sustainable biotechnological processes.

One of the key advantages of *Y. lipolytica* is its capacity for intensive lipid accumulation, including triacylglycerols, which can serve as reservoirs of precursors for isoprenoid biosynthesis. Additionally, these yeasts can accumulate carotenoids in lipid bodies, enhancing their stability and facilitating extraction [19]. The mevalonate pathway in *Y. lipolytica* shows high activity and efficiently generates intermediates for isoprenoid biosynthesis. This makes the yeast a safe and effective model organism for producing natural pigments and antioxidants. Besides its ability to accumulate triacylglycerols, *Y. lipolytica* also shows considerable potential for enhanced phospholipid biosynthesis, including phosphatidylcholine (PC) and phosphatidylserine (PS). Studies have shown that overexpressing genes such as *CDS*, *OPI3*, or *DGK1* can increase phospholipid production severalfold. This effect is especially pronounced when cells are cultivated on inexpensive and renewable carbon sources such as waste glycerol [20]. Furthermore, enzymes such as phosphatidate phosphatase Pah1 and phosphatidylserine synthase Pss1 play key roles in regulating phospholipid biosynthesis and maintaining balance between phospholipids and triacylglycerols in *Y. lipolytica* cells [21,22]. This property, combined with the ability of lycopene to integrate into cellular membranes, provides a promising basis for the development of *Y. lipolytica* strains capable of highly efficient lycopene biosynthesis.

*Y. lipolytica* yeasts also exhibit the ability to efficiently utilize industrial waste materials, including short-chain fatty acids (SCFAs), as alternative and low-cost carbon sources for biotechnological processes [23]. This microorganism can metabolize various short-chain fatty acids (SCFAs), including acetic, propionic, and butyric acids. These compounds are converted into acetyl-CoA, a key precursor for the biosynthesis of lipids, organic acids, and carotenoids [24,25]. Due to its high tolerance to environments with variable pH and salinity, *Y. lipolytica* can efficiently grow on SCFA mixtures derived from organic waste fermentation, making it an attractive organism within the concept of a circular bioeconomy [25,26].

Short-chain fatty acids are produced during anaerobic fermentation of organic matter and are recognized as valuable platform chemicals that can serve as precursors for the production of a wide range of bioproducts [27,28,29]. SCFAs are naturally generated in many environments, from landfills to wastewater treatment plants. Under controlled conditions, the formation of SCFAs contributes to biogas production. However, their uncontrolled release into the environment increases biological and chemical oxygen demand and leads to unpleasant odors [29,30]. Therefore, developing efficient methods for their utilization is a crucial direction in environmental protection and sustainable biotechnology.

The utilization of SCFAs by *Y. lipolytica* offers a solution that combines ecological and economic advantages. It not only reduces the negative environmental impact of these compounds but also enables their conversion into valuable biotechnological products, including lipids and carotenoids. Thus, the use of SCFAs as an inexpensive and renewable carbon source can significantly lower bioproduction costs while supporting a sustainable lycopene biosynthesis process in *Y. lipolytica*.

## 2. Results and Discussion

**Figure 1:**
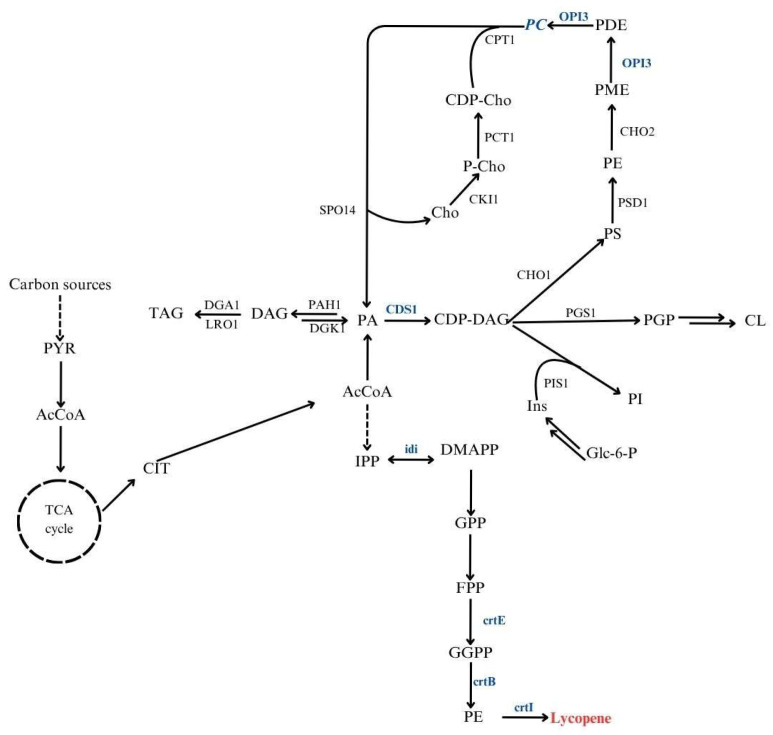
Integrated metabolic pathways linking lipid metabolism and lycopene biosynthesis in *Y. lipolytica***.** PYR, pyruvate; AcCoA, acetyl-CoA; CIT, citrate; PA, phosphatidic acid; DAG, diacylglycerol; TAG, triacylglycerol; CDP-DAG, cytidine diphosphate-diacylglycerol; PS, phosphatidylserine; PE, phosphatidylethanolamine; PC, phosphatidylcholine; PI, phosphatidylinositol; PGP, phosphatidylglycerophosphate; CL, cardiolipin; IPP, isopentenyl pyrophosphate; DMAPP, dimethylallyl pyrophosphate; GPP, geranyl pyrophosphate; FPP, farnesyl pyrophosphate; GGPP, geranylgeranyl pyrophosphate; PE, phytoene. Marked in blue, genes overexpressed in *Y. lipolytica* used in this study.

The oleaginous yeast *Y. lipolytica* is a robust platform for carotenoid production, including lycopene. Its flexible metabolism and strong lipid-accumulating capacity make it particularly suitable for this purpose [31]. Redirecting metabolic fluxes toward lipid biosynthetic pathways has been shown to improve carotenoid yields, emphasizing the connection between lipid metabolism and the efficiency of these processes [32]. Moreover, carotenoids such as lycopene can integrate into membrane phospholipids, enhancing membrane stability and oxidative resistance [10,11]. Engineering strains with improved phospholipid biosynthesis could therefore promote intracellular lycopene accumulation. Figure 1 presents a schematic overview of the central carbon metabolism and the interconnected lipid and isoprenoid biosynthetic pathways leading to lycopene formation in *Y. lipolytica*.

Another factor influencing production efficiency is the genomic context of integrated genes. The “position effect” can cause variation in transcription levels depending on the integration site, which is particularly relevant for multi-gene pathways such as lycopene biosynthesis [33]. Integrating the entire gene cluster within transcriptionally active genomic regions may ensure coordinated expression and improved pathway performance. Finally, the use of low-cost renewable substrates, including SCFAs, offers a sustainable route for lycopene production. *Y. lipolytica* efficiently converts SCFAs into acetyl-CoA and related intermediates, which can feed both lipid and carotenoid synthesis.

### 2.1. Construction and Molecular Characterization of Y. lipolytica Strains

Two *Y. lipolytica* strain backgrounds were analyzed in this study—a reference strain and a strain engineered for enhanced phospholipid biosynthesis—to examine how membrane composition and lipid metabolism influence lycopene accumulation.

To introduce the lycopene biosynthetic pathway, three different gene delivery systems were compared (see Section 3 for details). In the first approach, the genes were integrated individually into the genome in separate constructs. The second system employed a replicative plasmid carrying all four genes simultaneously, enabling multi-gene expression from a single vector. The third approach was based on the Golden Gate Assembly (GGA) method, in which three genes were introduced together while the fourth gene (*IDI*) was delivered separately.

This experimental design allowed the comparison of two host backgrounds and three expression strategies, providing insight into how lipid metabolism and vector architecture affect lycopene biosynthesis efficiency in *Y. lipolytica*.

**Figure 2:**
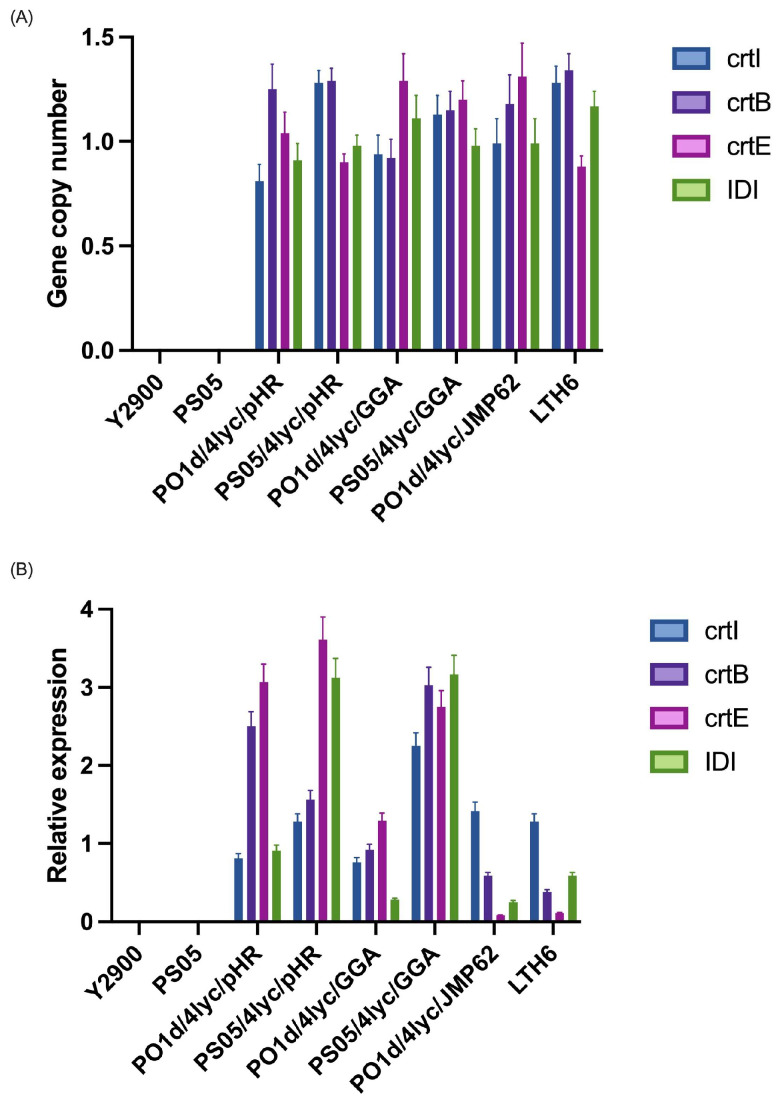
Gene copy number (**A**) and relative expression (**B**) of lycopene biosynthetic genes in *Y. lipolytica* strains carrying different expression systems.

The copy number of the four lycopene biosynthetic genes (*crtI*, *crtB*, *crtE*, and *IDI*) was quantified by qPCR using actin as a single-copy reference gene (Figure 2A). No amplification was detected in the parental strains *Y. lipolytica* Y2900 and PS05, confirming the absence of the target genes in the wild-type backgrounds. For strains harbouring the replicative plasmid (pHR system), the average copy number for all genes was close to one, indicating the presence of a single plasmid per cell on average, with minor variation between genes and host backgrounds. Strains constructed using the GGA system, in which three genes were introduced together and *IDI* separately, showed similar gene dosage, consistent with stable chromosomal integration of the constructs. The strains constructed with the integrated JMP62 vectors, where all four genes were independently inserted into the genome, exhibited copy number values ranging between 0.9 and 1.4. These results correspond to single-copy genomic integration events, as small deviations from 1.0 fall within the expected technical variation of qPCR.

Furthermore, the expression levels of *crtI*, *crtB*, *crtE*, and *IDI* were analyzed by RT-qPCR and normalized to the actin gene (Figure 2B). No detectable transcripts were found in the control strains (Y2900 and PS05), confirming the absence of the lycopene pathway prior to transformation. In strains containing the replicative plasmid (pHR), transcript levels were the highest overall, although not uniform across all four genes. Typically, two genes within the plasmid showed stronger expression, while the remaining two were less active. Interestingly, the pattern was not identical between the two genetic backgrounds: in the PS05 background, *crtE* expression approached that of *IDI*, while in the PO1d derivative, the highest expression was observed for the *crtB* and *crtE* genes, suggesting that host metabolic differences can modulate promoter activity even in the same vector context. The non-uniform expression observed in the pHR system may result from transcriptional interference between adjacent promoter regions and variation in plasmid supercoiling, both of which can alter local accessibility to the transcription machinery. Such position-dependent effects are frequently observed in yeast multi-gene plasmids, even when identical promoters are employed for each expression cassette. The GGA system, in which *crtI*, *crtB*, and *crtE* were placed on a single construct and *IDI* was integrated separately, resulted in a more balanced expression of the three co-located genes. The independently integrated *IDI* gene, however, showed lower expression in the PO1d derivative, while in the PS05 background, its level was comparable to the other genes, again pointing to background-dependent effects on transcriptional efficiency. For the integrative constructs, where each gene was inserted independently and randomly into the genome, expression levels were markedly lower and varied widely among genes. This variability is consistent with positional effects arising from random chromosomal integration, which can place each cassette in genomic regions of differing transcriptional activity.

### 2.2. Biomass Production

**Figure 3:**
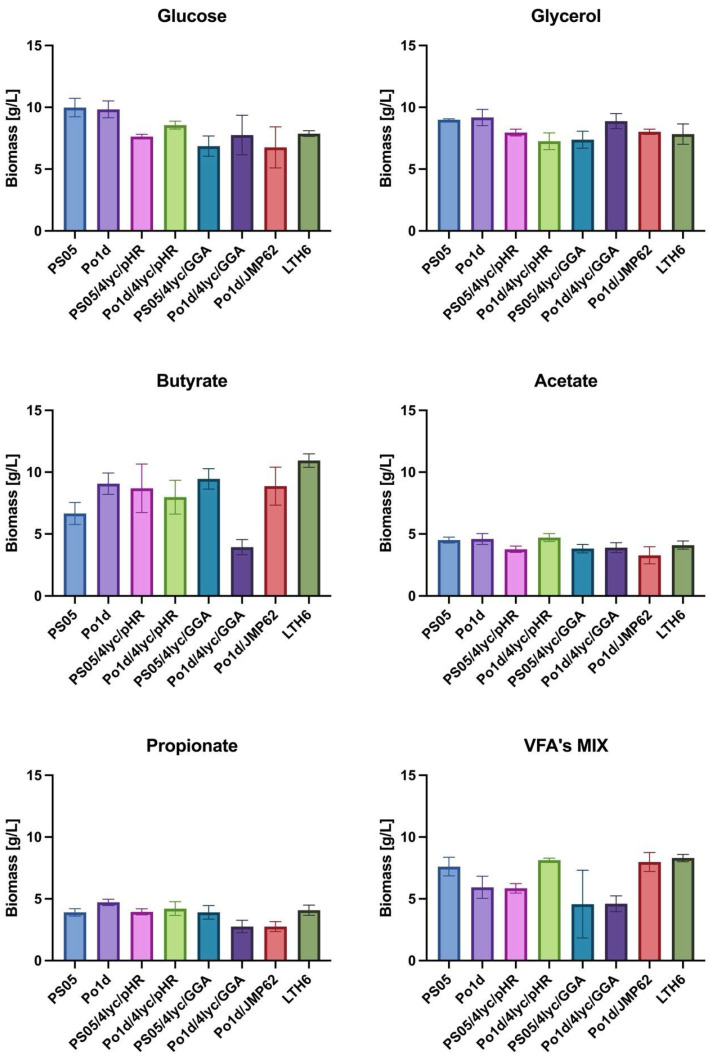
Biomass production of *Y. lipolytica* transformants cultivated in YNB medium with different carbon sources.

Analysis of biomass production (Figure 3) revealed that both the type of carbon source and the genotype of *Y. lipolytica* strains had a significant impact on growth efficiency. Glucose and glycerol proved to be neutral substrates for lycopene-producing strains, as the obtained biomass yield ranged from 6.9 to 10.0 g/L. Among all tested substrates, the highest biomass accumulation was observed during cultivation on butyrate, particularly for strain LTH6, which reached 10.9 ± 0.5 g/L. Other genetically modified strains, PS05/4lyc/pHR, Po1d/4lyc/pHR, PS05/4lyc/GGA and Po1d/JMP62, also exhibited relatively high biomass yields on this substrate, indicating its good metabolization by *Y. lipolytica*. Only strain Po1d/4Lyc/GGA showed a significantly reduced biomass yield.

In contrast to butyrate, cultures grown on acetate or propionate showed considerably lower growth performance—not exceeding 5 g/L—suggesting higher energetic costs and potential redox imbalance associated with the metabolism of these substrates. These findings are consistent with previous reports on *Y. lipolytica*. Efficient utilization of SCFAs by this yeast requires metabolic adaptation, activation of the β-oxidation pathway, and improved regulation of oxidative stress [34].

Interestingly, cultivation of certain strains (PO1d/4Lyc/pHR, PO1d/JMP62, LTH6) in a mixture of SCFAs (VFA’s MIX) resulted in biomass levels comparable to those achieved with glycerol. This may be attributed to a synergistic effect between different acids present in the medium, as previously reported for mixed carbon sources in *Y. lipolytica* [35]. This suggests that complex carbon sources can promote a more balanced metabolic flux, thereby supporting more efficient carbon assimilation and cell growth.

Furthermore, as demonstrated by Yun and colleagues [36] and Jing and colleagues [37], metabolic modifications can enhance yeast tolerance to demanding cultivation conditions and improve energy utilization efficiency. The obtained results confirm that both genetic engineering and carbon source optimization play crucial roles in improving biomass yield and precursor availability for carotenoid biosynthesis.

### 2.3. Lycopene Biosynthesis

**Figure 4:**
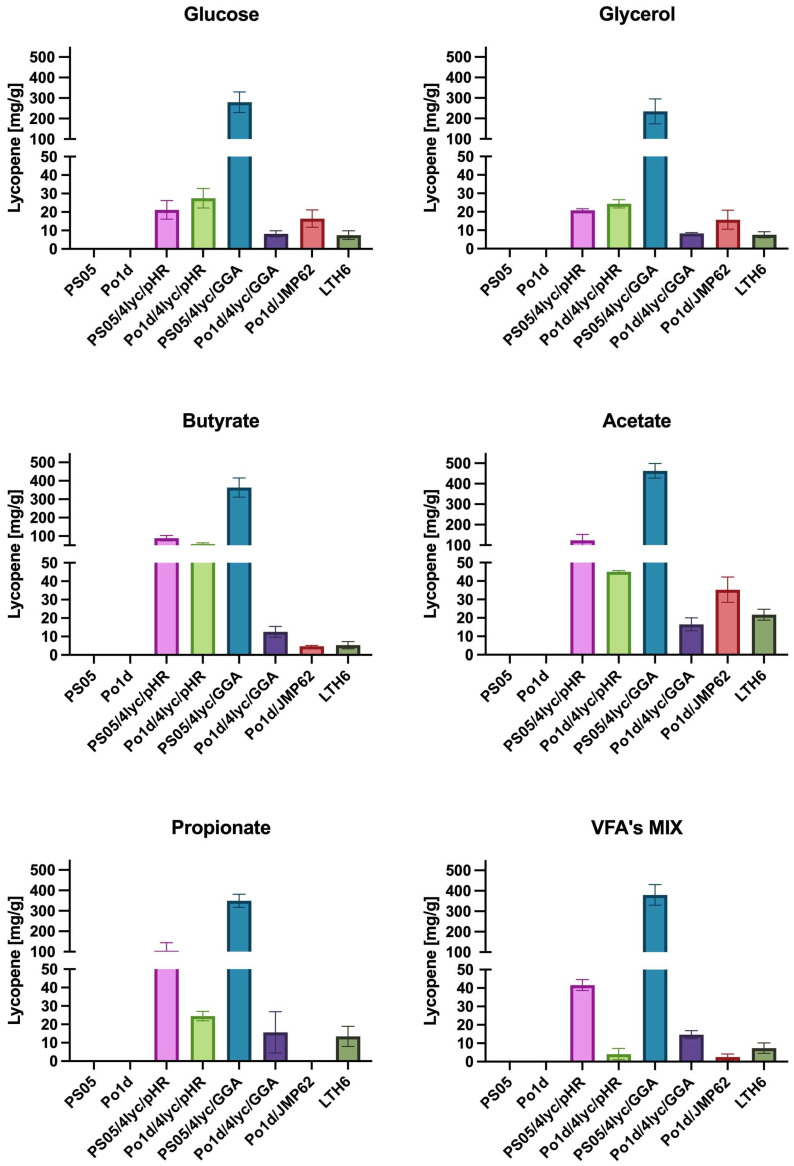
The effect of different carbon sources in YNB medium on lycopene production by *Y. lipolytica* transformants.

The obtained results clearly indicate that among the analyzed constructs, the *Y. lipolytica* strain PS05/4lyc/GGA exhibits the highest capacity for lycopene biosynthesis under all tested conditions (Figure 4). The maximum yield of 462.9 mg/g DCW on acetate and 379.9 mg/g DCW on the SCFA mixture exceeds the values reported so far for *Y. lipolytica*. For comparison, previous studies reported lycopene contents of 121 mg/g DCW [38] and 21.1 mg/g DCW [39] for *Y. lipolytica.* Record titers of 17.6 g/L were achieved only in highly optimized bioreactor fermentations, where the yield reached about 313 mg/g DCW [40]. This demonstrates that the presented genetic design enables very high production efficiencies to be achieved under simple, flask-scale laboratory conditions, confirming the effectiveness of the adopted engineering strategy.

Unlike most previous studies, the present work did not aim to maximize flux through the mevalonate pathway by repeatedly integrating multiple gene copies [38]. Instead, we focused on stable integration of the entire carotenoid biosynthetic module into a single genomic locus, combined with a background of enhanced phospholipid biosynthesis. This approach simplifies genetic construction, ensures stable expression, and allows high productivity without extensive process optimization. The results indicate that simultaneous activation of phospholipid and carotenoid biosynthesis produces a synergistic effect—an increased amount of cellular membranes and lipid structures (Figure S1) favors the accumulation of hydrophobic lycopene. This observation is consistent with the findings of Luo and colleagues [41], who demonstrated a positive correlation between intracellular lipid content and isoprenoid accumulation.

Particularly noteworthy are the results obtained for waste-derived substrates such as acetate and butyrate, which in this study supported higher lycopene production than conventional carbon sources like glucose or glycerol. The utilization of SCFAs enhances carbon flux through the β-oxidation pathway and increases acetyl-CoA formation—the key precursor for mevalonate and carotenoid biosynthesis [42]. This observation is further supported by studies on *Rhodotorula glutinis* treated with sodium butyrate [43], where enhanced carotenoid biosynthesis was linked to the regulation of membrane stability and redistribution of acetyl-CoA in response to oxidative stress. The use of such substrates not only reduces production costs but also aligns with the concept of sustainable biotechnology and a circular bio-economy, distinguishing this approach from earlier strategies based on pure sugars or isoprenol [39,41].

Differences observed between the PS05 and Po1d strains indicate that the genetic background of the host plays a crucial role in the efficiency of multi-gene construct expression. Strains with enhanced phospholipid biosynthesis showed not only higher lycopene accumulation but also greater metabolic stability during cultivation. In contrast, the reduced productivity observed for Po1d/4lyc/GGA in prolonged cultures may indicate metabolic overload or a disturbance in redox balance. This finding is consistent with the observations of Ma and colleagues [40], who reported the sensitivity of *Y. lipolytica* to excessive flux through the carotenoid pathway. Differences in biomass color among *Y. lipolytica* cultures grown on different substrates are depicted in Figure S2.

When lycopene titers obtained in this study are expressed per liter of culture (Table 1), the PS05/4lyc/GGA strain produced up to 3.41 ± 0.18 g/L on butyrate, which exceeds some of the previously reported values for *Y. lipolytica.* For instance, Schwartz and colleagues [39] achieved approximately 0.21 g/L (21.1 mg/g DCW), while Luo and colleagues [38] reported around 5.1 g/L (121 mg/g DCW) under optimized metabolic conditions. The strain described by Luo and colleagues [41], which combined the native mevalonate pathway with the isopentenol utilization pathway (IUP) and enhanced intracellular hydrophobicity, reached 4.2 g/L lycopene, but only after extensive process optimization in a controlled bioreactor system. Even in the most advanced study by Ma and colleagues [40], where removal of lycopene-cyclase substrate inhibition enabled β-carotene titers of 39.5 g/L, the maximum lycopene concentration of 17.6 g/L was obtained exclusively under fed-batch bioreactor conditions and at significantly higher cell densities than those used here. In shake-flask systems, parameters such as aeration, pH, nutrient gradients, and oxygen transfer rate are not precisely regulated, which can limit both cell growth and metabolite accumulation. Future studies should therefore focus on scaling up the process under controlled bioreactor conditions, where optimized aeration and feeding strategies may further enhance lycopene yields and metabolic stability. Nonetheless, the 3.4 g/L lycopene titer achieved in this work under simple flask cultivation demonstrates productivity comparable to bioreactor-scale systems, confirming the efficiency of the single-locus genomic integration strategy combined with enhanced phospholipid biosynthesis.

The remarkable performance of the PS05/4lyc/GGA strain also highlights the advantage of using short-chain fatty acids as carbon sources. Both butyrate and acetate supported higher lycopene titers than conventional substrates such as glucose or glycerol, indicating that SCFAs promote a more efficient metabolic flux toward acetyl-CoA—the central precursor of the mevalonate pathway. This observation is consistent with previous reports linking increased lipid biosynthesis and intracellular hydrophobicity with improved isoprenoid accumulation [41].

In contrast, strains derived from the Po1d background, which lack the enhanced phospholipid biosynthetic capacity, displayed significantly lower titers (typically below 0.5 g/L). These differences confirm that both the host background and the integration strategy play a crucial role in determining the efficiency of carotenoid pathway expression. The combination of single-locus genomic integration, co-activation of phospholipid biosynthesis, and utilization of waste-derived SCFAs thus enables lycopene productivities that approach or even rival those achieved in highly optimized bioreactor systems, while maintaining the simplicity and sustainability of flask-scale cultivation.

Recent developments in lycopene production using *Y. lipolytica* are summarized in Table 2.

### 2.4. Lipid Biosynthesis

**Figure 5:**
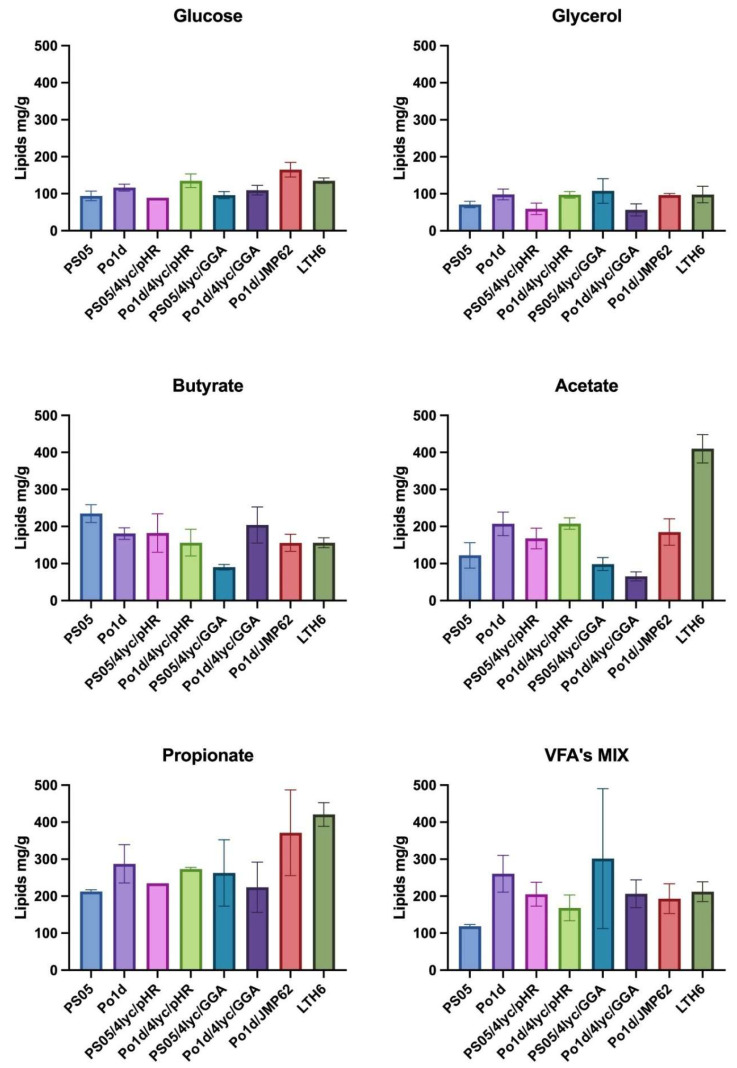
The effect of different carbon sources in YNB medium on lipids production by *Y. lipolytica* transformants.

The lipid accumulation profiles of the analyzed *Y. lipolytica* strains highlight a close link between lipid metabolism, phospholipid biosynthesis, and carotenoid formation. The PS05/4lyc/GGA strain, which showed the highest lycopene productivity, maintained only moderate lipid levels (90–263 mg/g) (Figure 5), suggesting that lipid flux was redirected from neutral lipid synthesis toward membrane phospholipid formation. A similar trend was reported by Szczepańska and colleagues [20], who observed reduced neutral lipid accumulation accompanying enhanced phospholipid biosynthesis in *Y. lipolytica*. This metabolic shift likely reflects a trade-off between storage lipid formation and the expansion of membrane structures that accommodate hydrophobic metabolites such as lycopene. The redirection of acetyl-CoA and NADPH toward phospholipid and isoprenoid synthesis therefore limits triacylglycerol formation while promoting carotenoid accumulation. Future transcriptomic or metabolomic studies could further clarify how this balance between membrane remodeling and lipid storage regulates carotenoid biosynthesis in *Y. lipolytica*.

In contrast, Po1d-based strains accumulated higher total lipid levels (up to 287 mg/g on propionate) (Figure 5), but this did not correspond with increased lycopene production. This imbalance suggests competition for acetyl-CoA and NADPH between fatty acid and carotenoid biosynthetic pathways, as observed in engineered *Y. lipolytica* strains by Naveira-Pazos and colleagues [44]. The introduction of the GGA construct into the Po1d background likely intensified this competition, reducing both lipid accumulation and carotenoid productivity compared to the PS05 background.

The effect of the carbon source on lipid metabolism was particularly pronounced. SCFAs such as butyrate, acetate, and propionate promoted higher lipid accumulation than conventional substrates like glucose or glycerol. This trend agrees with Wang and colleagues [45], who demonstrated that SCFAs serve as efficient carbon sources for lipid synthesis in *Y. lipolytica*, providing a direct route to acetyl-CoA via β-oxidation and thereby supporting both lipid and secondary metabolite biosynthesis.

The highest lipid contents for PS05/4lyc/GGA (Figure 5) were observed on the SCFA mixture (≈302 mg/g) and on propionate (≈263 mg/g), conditions favoring enhanced acetyl-CoA generation and fatty acid turnover. A comparable effect of mixed or waste-derived substrates was previously described by Kot and colleagues [46], who showed that complex carbon sources stimulate both lipid storage and secondary metabolism in *Y. lipolytica*.

## 3. Materials and Methods

### 3.1. Microorganisms

The *Y. lipolytica* strains used in this study are listed in Table 3. Additionally, *Escherichia coli* DH5α was used for plasmid construction and amplification.

To recover prototrophy (if necessary), strains were transformed with a purified *I-Sce-I* fragment of the plasmid *JMP62(URA3ex* or *LEU2ex*) that contained the necessary gene [50].

### 3.2. Genes

To construct lycopene-producing strains, four heterologous genes from *Pantoea agglomerans* were utilized: *crtI* (*phytoene desaturase*—*PD*), *crtB* (*phytoene synthase*—*PS*), *crtE* (*geranylgeranyl diphosphate synthase*—*GGDS*), and *idi* (*isopentenyl diphosphate isomerase*—*IDI*). The coding sequences of these genes were codon-optimized for expression in *Y. lipolytica* using the Benchling (www.benchling.com) Codon Optimization Tool based on *Y. lipolytica* codon usage preferences.

### 3.3. Plasmids’ and Y. lipolytica Transformants’ Construction

Three types of plasmid systems were employed for gene expression: JMP62-*URA3ex(LEU2ex)-pTEF* [50], *pHR_XPR2_hrGFP* [48], and plasmids designed for the Golden Gate Assembly (GGA) cloning method [49].

In the first type of *Y. lipolytica* transformants, the *JMP62URA3ex(LEU2ex)-pTEF* plasmid was used, carrying a single gene per construct. Four different plasmids were prepared and sequentially integrated into the *Y. lipolytica* genome. Expression cassettes were excised using the *NotI* restriction enzyme and integrated into the yeast genome through Zeta retrotransposable sequences. After integration of the first two genes (*crtI* and *crtB*) into the genome, the Cre recombinase system was applied to excise the selectable markers flanked by lox sites, thereby enabling marker recycling for subsequent transformations. Because integration occurred randomly, multiple transformants were screened, and one was selected for further analysis. The sequences of primers used for gene amplification are listed in Table S1.

The second type of transformants was constructed using a modified pHR_XPR2_hrGFP plasmid [48], which contains a *Y. lipolytica* centromeric sequence and functions as a replicative vector (without restriction digestion, carrying the *URA3* marker). Four genes were inserted into this plasmid simultaneously using the Gibson Assembly method and synthetic DNA fragments containing the *TEF* promoter, the gene of interest, and the *LIP2* terminator. The sequences of these synthetic constructs are provided in the Supplementary Materials (pHR-related sequences). The resulting transformants were subsequently transformed with an I-SceI–digested *LEU2ex* genetic marker to restore prototrophy. After screening multiple transformants, one representative strain was selected for further analysis.

The lycopene biosynthetic pathway was assembled using the Golden Gate Assembly (GGA) system, specifically optimized for *Y. lipolytica* [49]. The cloning followed a hierarchical modular structure comprising Level 0, Level 1, and Level 2 assembly steps. In the Level 0 stage, individual standardized parts—promoters, coding sequences, and terminators—were synthesized and cloned into pYTK-compatible entry vectors flanked by BsaI recognition sites, generating specific four-base overhangs according to the *Y. lipolytica* toolkit standard. During Level 1 assembly, single transcriptional units were created by ligating the constitutive *TEF* promoter, the codon-optimized gene of interest (*crtI*, *crtB*, *crtE*, or *IDI*), and the *LIP2* terminator using BsaI. Each transcriptional cassette was cloned into a destination vector containing either the *URA3* selection marker. In the Level 2 assembly, three expression cassettes (*crtI*, *crtB*, and *crtE*) were combined into a single multigene plasmid with *LEU2* selection marker using BsmBI-mediated digestion–ligation. *IDI* was assembled independently in a separate Level 1 plasmid. Both plasmids were designed for genomic integration and contained flanking Zeta retrotransposon sequences to enable random chromosomal integration upon linearization with NotI.

Multiple transformants were screened, and one was selected for subsequent experiments. The majority of the transformants showed similar lycopene levels, and the selected clone exhibited the characteristic lycopene content representative of the group.

**Figure 6:**
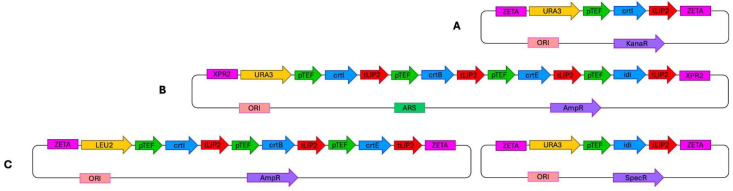
Plasmid constructs used for the introduction of the lycopene biosynthetic pathway into the *Y. lipolytica* genome. (**A**) Integration-type plasmid *JMP62-pTEF* (example shown with the *crtI* gene and *URA3ex* marker). (**B**) Replicative vector *pHR* carrying all four cloned genes. (**C**) Golden Gate Assembly system comprising plasmids containing three genes (*crtI*, *crtB*, *crtE*) and one gene (*IDI*), respectively.

All constructs were verified using PCR and sequencing (Genomed S.A, Warsaw, Poland). The overall design and organization of the three lycopene biosynthetic pathway constructions are illustrated in Figure 6.

### 3.4. Cloning and Transformation Procedures

All restriction enzymes, Phusion High-Fidelity DNA Polymerase, and T4 DNA Ligase were obtained from Thermo Scientific (Waltham, MA, USA) and used according to the manufacturer’s protocols. Plasmid DNA was isolated from *E. coli* cultures using the Plasmid Mini Kit (A&A Biotechnology, Gdańsk, Poland). DNA fragments recovered from agarose gels were purified with the Gel Out Extraction Kit (A&A Biotechnology, Gdańsk, Poland).

Chemically competent *E. coli* DH5α cells were transformed using the standard heat-shock method and subsequently selected on LB agar plates containing the appropriate antibiotic, while *Y. lipolytica* transformations were performed via the lithium acetate procedure as previously described [47]. Transformants were selected on YNB-Leu, YNB-Ura, YNB or YG-Hygro (Yeast Extract, Glucose) plates, depending on the selectable marker present in the construct. All transformants were verified by PCR to confirm the correct integration of the expression cassettes.

### 3.5. Reverse Transcription and Quantitative RT-PCR

Genomic DNA was isolated following the method of Hoffman and Winston [51]. Total RNA was extracted using TRIzol™ Reagent (Invitrogen, Carlsbad, CA, USA) according to the manufacturer’s protocol. The quantity and quality of the nucleic acids were assessed using an ND-1000 spectrophotometer (NanoDrop, Wilmington, DE, USA). Complementary DNA (cDNA) was synthesized from the RNA templates with the Maxima First Strand cDNA Synthesis Kit for RT-qPCR (Thermo Fisher Scientific, Waltham, MA, USA) following the supplier’s protocol, and the obtained cDNA was purified using the QIAquick™ Nucleotide Removal Kit (QIAGEN, Venlo, The Netherlands).

Quantitative real-time PCR (qRT-PCR) analyses were performed with the Maxima SYBR Green qPCR Master Mix (2×) (Thermo Fisher Scientific, Waltham, MA, USA) in a total reaction volume of 10 µL. Each reaction contained 0.5 µM of forward and reverse primers and 1 µg of template DNA (cDNA or genomic DNA). Amplifications were carried out on a CFX Connect Real-Time PCR Detection System (Bio-Rad, Hercules, CA, USA) using the following thermal profile: initial denaturation at 95 °C for 5 min, followed by 40 cycles of 95 °C for 10 s, 55 °C for 10 s, and 72 °C for 10 s. Fluorescence signals were recorded at the end of each extension step, and product specificity was confirmed by melting curve analysis at the end of the run. The expression levels of the analyzed genes were determined using primer pairs listed in Table S1. Relative expression levels were calculated using the method described by Schmittgen and Livak [52].

### 3.6. Flask Cultivation

One transformant of each strain was tested (Po1d and PS05 derivatives). The preculture was conducted in 50 mL of the appropriate YNB medium (corresponding to the analyzed carbon source) at 28 °C with 180 rpm of agitation for 48 h. After that time, cells were washed three times with sterile distilled water and used for inoculation. The initial OD_600_ of the production culture was set at 0.5 for each strain.

Shake-flask cultivations were performed in minimal YNB-based medium whose composition is summarized in Table 4. For the mixture of short-chain fatty acids (SCFA’s MIX), the medium contained a total carbon concentration of 32 g/L, consisting of 6.5 g/L sodium acetate, 7.5 g/L sodium propionate, and 18 g/L sodium butyrate (all from Sigma-Aldrich, Saint Louis, MO, USA), corresponding to a C/N ratio of 60. Composition was developed and optimized in previous studies by Kupaj [42].

Cultures were carried out in 250 mL Erlenmeyer flasks (without baffles) containing 50 mL of medium at 28 °C and 180 rpm for 120 h. Each experiment was conducted in three independent biological replicates. For PO1d and PS05 strains, all media were supplemented with 0.1 g L^−1^ of uracil and leucine.

### 3.7. Dry Biomass Determination

After incubation, the entire culture was transferred into Falcon tubes and adjusted to a final volume of 50 mL with distilled water. Subsequently, 40 mL of the culture was centrifuged at 5000 rpm for 5 min using a centrifuge model 5804/5804 R (Eppendorf, Hamburg, Germany). One milliliter of the supernatant was collected for HPLC analysis of the residual carbon source using a Thermo Scientific system (Waltham, MA, USA) equipped with a HyperRez Carbohydrate H^+^ column (Thermo Scientific), a UV detector (λ = 210 nm; Dionex, Sunnyvale, CA, USA), and a refractive index detector (Shodex, Ogimachi, Japan). The column was eluted with 25 mM trifluoroacetic acid at 35 °C and a flow rate of 0.6 mL/min. The remaining supernatant was discarded. The biomass was washed with distilled water, lyophilized, and weighed to determine the dry cell weight (DCW).

### 3.8. Carotenoids Extraction and Quantification

Lycopene concentration in the biomass was determined using a modified extraction protocol based on the method described by Schwartz and colleagues. Briefly, 5 mL of the culture was transferred into dark 15 mL Falcon tubes and centrifuged at 1500 rpm for 5 min. The biomass was washed twice with distilled water, and the supernatant was discarded. Subsequently, 3 M HCl was added to the pellet, and the tubes were incubated in a thermoblock at 100 °C for 3 min with agitation at 1000 rpm, followed by immediate cooling on ice for 3 min. After cooling, the samples were centrifuged to remove residual acid. Acid-washed glass beads and 5 mL of chloroform were then added, and the mixture was shaken at 2500 rpm for 2 h at 3 °C to extract lycopene. Finally, the samples were centrifuged, and 1 mL of the colored supernatant was collected for further analysis.

Carotenoids were quantified using an HPLC system (UltiMate 3000, Thermo Scientific, USA) equipped with a YMC Carotenoid column (5 μm particle size, YMC Co., Kyoto, Japan). The mobile phase consisted of Eluent A (81% methanol, 15% tert-butyl methyl ether, 4% water) and Eluent B (6% methanol, 90% tert-butyl methyl ether, 4% water). The flow rate was set to 1.0 mL/min, and the gradient program involved a linear increase from 1% to 100% of Eluent B over 90 min. Carotenoids were detected at 450 nm using a UV–Vis detector. The retention time for lycopene was approximately 72 min (Figure S3), and its concentration was determined using a calibration curve generated from standard solutions of the pure compound.

### 3.9. Lipid Extraction and Quantification

The lyophilized biomass was processed for fatty acid extraction and derivatization to fatty acid methyl esters (FAMEs) using a previously described method. Briefly, 10 mg of freeze-dried biomass was mixed with 2 mL of a solvent solution consisting of 2.5% H_2_SO_4_ and 97.5% methanol, with 50 μg/mL of C17:0 as an internal standard, in Pyrex glass tubes (Sigma-Aldrich, Saint Louis, MI, USA). The samples were thoroughly mixed and incubated overnight at 80 °C to form FAMEs.

FAMEs were extracted using hexane and 0.9% NaCl. The organic phase containing FAMEs was collected and stored at −20 °C until analysis. FAME analysis was performed using gas chromatography coupled to an FID detector using a Shimadzu instrument (Kyoto, Japan) equipped with a Zebron ZB-FAME capillary column (30 m × 0.25 mm × 0.20 μm). Samples (1 μL) were injected in splitless mode at 250 °C, with hydrogen as the carrier gas at a flow rate of 1 mL/min. Fatty acid identification was performed by comparing retention times with those of FAME mixture known standards (Sigma-Aldrich, Saint Louis, MI, USA).

## 4. Conclusions

This study demonstrates the successful engineering of *Y. lipolytica* as an efficient microbial platform for lycopene biosynthesis using short-chain fatty acids as alternative carbon sources. The integration of the lycopene biosynthetic genes (*crtE*, *crtB*, *crtI*) from *P. agglomerans* into a single genomic locus, together with overexpression of the *IDI* gene and enhanced phospholipid biosynthesis, resulted in exceptionally high lycopene yields. Under simple flask cultivation conditions, the strain produced up to 462.9 mg/g DCW and 3.41 g/L. These values are comparable to or even exceed those previously reported for bioreactor-scale fermentations.

The results highlight that phospholipid pathway activation creates a favorable intracellular environment for hydrophobic carotenoid accumulation, while the use of SCFAs such as acetate and butyrate promotes efficient carbon flux toward acetyl-CoA and the mevalonate pathway. Furthermore, the genetic background of the host strain plays a crucial role in determining productivity, as strains with enhanced membrane lipid biosynthesis showed both higher lycopene accumulation and improved metabolic stability. This work confirms the feasibility of coupling waste-derived substrates with metabolic engineering of *Y. lipolytica* to achieve sustainable, low-cost, and high-yield microbial production of lycopene. Future research should focus on scaling up this process and optimizing fermentation parameters to further advance the development of environmentally friendly biotechnological production platforms.

## Figures and Tables

**Table 1:** Lycopene production (g/L) by various *Y. lipolytica* transformants cultivated on different carbon sources.

Carbon Sources	Strain							
	PS05	Po1d	PS05/4lyc/pHR	Po1d/4lyc/pHR	PS05/4lyc/GGA	Po1d/4lyc/GGA	Po1d/JMP64	LTH6
Glucose	0	0	0.16 ± 0.04	0.24 ± 0.05	1.94 ± 0.50	0.06 ± 0.01	0.11 ± 0.03	0.06 ± 0.02
Glycerol	0	0	0.17 ± 0.00	0.18 ± 0.03	1.70 ± 0.31	0.07 ± 0.01	0.13 ± 0.04	0.06 ± 0.02
Butyrate	0	0	0.77 ± 0.19	0.45 ± 0.08	3.41 ± 0.18	0.05 ± 0.02	0.04 ± 0.01	0.06 ± 0.02
Acetate	0	0	0.47 ± 0.12	0.21 ± 0.02	1.71 ± 0.17	0.06 ± 0.01	0.12 ± 0.40	0.09 ± 0.02
Propionate	0	0	0.41 ± 0.16	0.1 ± 0.02	1.35 ± 0.11	0.04 ± 0.02	0	0.05 ± 0.02
SCFA’s MIX	0	0	0.24 ± 0.02	0.03 ± 0.03	1.69 ± 1.02	0.07 ± 0.02	0.02 ± 0.01	0.06 ± 0.02

**Table 2:** Developments in lycopene production using *Y. lipolytica*.

Strain	Strategy	Substrate	Yield (mg/g)	Titer (g/L)	Conditions	Reference
PS05/4lyc/GGA	Enhanced phospholipid biosynthesis and genomic integration of lycopene pathway genes via Golden Gate plasmids	Glucose (YNB)	280	1.94	Flask culture	This study
PS05/4lyc/GGA		Acetate (YNB)	463	1.71	Flask culture	This study
PS05/4lyc/GGA		Butyrate (YNB)	363	3.41	Flask culture	This study
A38-3	Employed different methods to improve the key genes overexpression including crtE, crtB, crtI, AtoB, HMGR, ERG12	Glucose/glycerol (YPD)	121	5.1	Bioreactor, fed-batch mode	[38]
HEBI HV8I	Overexpressed key genes HMG1, MVD1, ERG8, and CrtI to enhance flux through the mevalonate and lycopene biosynthetic pathways	Glucose (YPD)	21.1	0.21	Bioreactor, fed-batch mode	[39]
YLMA34	Released the substrate inhibition of lycopene cyclase	Glucose (YPD)	313	17.6	Bioreactor, fed-batch mode	[40]
YLlyc-9	Synthesized carotenoid precursors IPP and DMAPP through the IUP	Yeast extract, glucose, palmitic acid, isoprenol (YNB)	175	4.2	Bioreactor, batch mode	[41]

**Table 3:** Strains of *Y. lipolytica* used in this study.

Strain Name	Genotype	Used Plasmid	Bibliography
PO1d	*MATa ura3-302 leu2-270 xpr2-322*	-	[47]
PS05	*MATa ura3-302 leu2-270 xpr2-322 pTEF-CDS pTEF-OPI3*	-	[20]
PO1d/4lyc/pHR	*PO1d: TEF-crtI; TEF-crtB; TEF-crtE; TEF-IDI*	pHR_XPR2_hrGFP [48]	This study
PS05/4lyc/pHR	*PS05: TEF-crtI; TEF-crtB; TEF-crtE; TEF-IDI*	pHR_XPR2_hrGFP [48]	This study
PO1d/4lyc/GGA	*PO1d: TEF-crtI; TEF-crtB; TEF-crtE; TEF-IDI*	Golden Gate Assembly plasmids [49]	This study
PS05/4lyc/GGA	*PS05: TEF-crtI; TEF-crtB; TEF-crtE; TEF-IDI*	Golden Gate Assembly plasmids [49]	This study
PO1d/JMP62	*PO1d: TEF-crtI; TEF-crtB; TEF-crtE; TEF-IDI*	JMP62TEF [50]	This study
LTH6	*PO1d: TEF-crtI; TEF-crtB; TEF-crtE; TEF-IDI*	JMP62TEF [50]	This study

**Table 4:** Composition of the YNB medium containing different carbon sources and a C/N ratio of 60, used for lycopene biosynthesis by *Y. lipolytica* transformants during flask cultivation.

Carbon Source		(NH_4_)_2_SO_4_	MgSO_4_ × 7H_2_O	YNB (w/o (NH_4_)_2_SO_4_ and aa)	Phosphate Buffer
g/L					
Glucose	30	0.94	1.0	1.7	0.05 M, pH 7.0
Glycerol	30	0.92			
Acetate	30	0.94			
Propionate	30	1.15			
Butyrate	30	1.28			
Mix SCFA (A/P/B)	32 (6.5/7.5/18)	1.26			

## Data Availability

The original contributions presented in this study are included in the article/Supplementary Materials. Further inquiries can be directed to the corresponding author.

## Supplementary Materials

The following supporting information can be downloaded at https://www.mdpi.com/article/10.3390/molecules30214321/s1. Table S1. List of primers used in this study. Figure S1. Morphology of *Y. lipolytica* strains (W29—wild type, PS05—phospholipid overproducing strain) growing in a YNB medium with glucose. Figure S2. Effect of carbon source and genetic constructs on pigmentation and it’s intensity in lycopene-producing *Y. lipolytica* transformants (order: PS05, PO1d, PS05/4lyc/pHR, PO1d/4lyc/pHR, PS05/4lyc/GGA, PO1d/4lyc/GGA, PO1d/JMP62, LTH6). Figure S3. HPLC chromatographic profiles of lycopene. (A) Extract from *Y. lipolytica* strain PS05/4lyc/GGA. (B) Reference chromatogram of lycopene standard used for identification.
