# Supplementary material for: Advances in Lycopene Production: From Natural Sources to Microbial Synthesis Using Yarrowia lipolytica
Source: Molecules. 2025 Nov 6;30(21):4321. doi: 10.3390/molecules30214321 (PMC12608319; doi:10.3390/molecules30214321)
Supplement: Supplementary file 1 [file molecules-30-04321-s001.zip › molecules-3948170-supplementary.pdf]

SUPPLEMENTARY MATERIALS

Advances in Lycopene Production: From Natural Sources to Microbial Synthesis Using *Yarrowia lipolytica*

Authors: Paweł Moroz [1], Aleksandra Bartusiak [1], Julia Niewiadomska [1], Kacper Szymański [1], Tomasz Janek [1], Anna Kancelista [1], Anna Gliszczyńska [2] and Zbigniew Lazar [1]

1- Departament of Biotechnology and Food Microbiology, Wroclaw University of Environmental and Life Sciences, Chelmonskiego 37, 51-630, Wroclaw, Poland
2- Department of Food Chemistry and Biocatalysis, Wroclaw University of Environmental and Life Sciences, Norwida 25, 50-375 Wroclaw, Poland

1. Synthetic genes

Sequences of the synthetic genes were ordered from GeneArt (Thermo Fisher Scientific) and were codon optimized for *Y. lipolytica* using algorithm available on Benchling platform (http://benchling.com/). All genes come from *Pantoea agglomerans.*

Sequences used for construction of Golden Gate plasmids

GGA-PS (*phytoene synthase - crtB)*

gcatCGTCTCATCGGTCTCAAATGTCCCAGCCTCCCTTGCTAGACCACGCCACTCAG
ACCATGGCAAACGGATCTAAATCTTTTGCCACGGCCGCTAAGTTGTTCGACCCCGC
TACCAGGAGATCGGTCCTGATGCTTTACACCTGGTGTCGACACTGTGACGACGTCA
TTGACGACCAGACCCATGGCTTCGCTTCCGAGGCCGCCGCTGAGGAGGAGGCTAC
CCAACGACTGGCCCGACTCCGTACCCTAACTCTGGCGGCTTTCGAGGGCGCGGAG
ATGCAGGACCCCGCCTTCGCGGCCTTTCAGGAAGTTGCACTTACCCACGGGATTAC
GCCTCGAATGGCTCTCGACCATCTGGATGGATTCGCCATGGACGTCGCTCAAACAC
GATACGTGACATTTGAAGATACTCTACGCTACTGCTATCACGTTGCCGGTGTGGTTG
GTCTGATGATGGCCAGAGTGATGGGTGTGCGAGATGAACGAGTGCTGGACAGAGC
CTGCGACCTTGGACTTGCCTTCCAGCTCACGAATATCGCTCGGGACATCATTGACG
ACGCCGCTATTGATCGTTGCTACCTGCCCGCTGAGTGGTTGCAAGATGCGGGCTTG
ACACCCGAGAACTACGCTGCCCGAGAAAACAGAGCTGCGCTCGCTCGAGTCGCT
GAGCGGCTGATTGACGCCGCTGAACCATACTACATTTCCAGTCAGGCTGGCCTACA
TGATTTGCCCCCCAGATGCGCCTGGGCCATTGCAACAGCGCGGAGTGTTTACCGAG
AAATCGGTATCAAGGTCAAAGCCGCTGGTGGATCGGCCTGGGACCGTAGACAGCA
CACCTCTAAGGGCGAAAAAATCGCCATGCTGATGGCCGCGCCGGGACAGGTCATT
CGGGCCAAGACAACAAGGGTCACGCCACGACCAGCCGGGCTTTGGCAGCGACCA
GTCTAATCTATGAGACCTGAGACGgcat

GGA-PD (*phytoene desaturase - crtI)*

gcatCGTCTCATCGGTCTCAAATGAAGAAAACAGTCGTAATCGGCGCCGGATTCGGT
GGCTTGGCTCTTGCAATTCGACTACAGGCGGCTGGTATTCCTACCGTCCTCCTGGA
GCAGCGGGATAAGCCTGGAGGCCGAGCCTATGTGTGGCACGACCAGGGATTCACT
TTCGACGCCGGACCCACGGTTATCACTGACCCTACTGCGCTGGAGGCTCTTTTCAC
ACTCGCTGGCCGGCGCATGGAAGATTACGTGAGACTGCTGCCTGTAAAGCCATTCT
ACCGACTGTGTTGGGAGTCTGGAAAGACTCTGGACTATGCCAACGATTCTGCTGA
GCTTGAAGCTCAAATCACTCAGTTTAACCCCAGGGATGTCGAGGGATACCGACGG
TTCCTTGCCTACTCTCAAGCAGTGTTCCAAGAGGGGTACCTGCGTCTGGGATCTGT
GCCCTTTCTTTCTTTTCGAGACATGCTTAGAGCTGGACCCCAGCTCTTGAAGCTGC
AGGCATGGCAATCCGTGTACCAATCGGTCAGCCGGTTCATCGAGGATGAGCATCTC
CGACAGGCATTTTCTTTCCACTCCCTTCTGGTGGGAGGAAACCCCTTCACTACTTC
CTCTATCTACACTCTAATCCACGCCCTGGAGCGAGAATGGGGCGTGTGGTTTCCCG
AGGGAGGTACCGGCGCGCTTGTTAACGGAATGGTGAAGCTCTTCACAGACTTGGG
CGGCGAGATAGAGCTTAATGCGCGAGTTGAAGAGCTGGTGGTAGCAGACAATAGA
GTATCCCAGGTCCGACTGGCCGATGGCCGAATCTTCGACACGGATGCAGTGGCTTC
TAACGCCGATGTGGTCAATACATATAAGAAGCTCCTGGGACACCACCCCGTCGGAC
AAAAGCGCGCCGCTGCCCTAGAGCGGAAGTCGATGTCCAACTCGCTGTTCGTTCT
GTACTTTGGACTGAACCAGCCCCATTCCCAGCTAGCTCATCACACCATCTGCTTTG
GCCCGCGGTACCGGGAGCTCATTGATGAGATCTTCACCGGGTCGGCTCTAGCCGAC
GATTTCTCTTTGTACCTTCATTCTCCCTGTGTTACCGACCCTTCCCTTGCCCCTCCCG
GGTGTGCCTCGTTCTATGTGCTCGCCCCTGTGCCTCATCTGGGTAACGCGCCTCTG
GATTGGGCCCAGGAGGGTCCCAAGCTCCGTGACCGTATTTTTGACTACCTGGAGG
AGCGATATATGCCCGGTCTGCGTTCTCAGCTGGTCACTCAACGAATCTTTACACCG
GCCGACTTCCACGACACCCTGGACGCTCACCTTGGATCGGCTTTCTCTATTGAACC
TCTCCTTACCCAGAGTGCCTGGTTTCGACCACATAACCGAGACTCGGATATTGCTA
ACCTGTATCTCGTCGGAGCTGGCACACATCCGGGAGCCGGCATTCCGGGAGTGGT
CGCTTCTGCAAAGGCTACTGCCTCGCTGATGATCGAGGACCTGCAATGATCTATGA
GACCTGAGACGgcat

GGA-GGDS (*geranylgeranyl diphosphate synthase - crtE)*

gcatCGTCTCATCGGTCTCAAATGGTGTCTGGATCTAAGGCAGGGGTGAGTCCTCAC
CGGGAGATTGAGGTGATGCGTCAGTCAATCGACGACCATCTGGCGGGGCTGCTGC
CCGAAACCGACAGCCAGGACATCGTGTCCCTGGCCATGCGAGAGGGCGTGATGGC
TCCCGGGAAGCGAATTCGGCCACTTCTGATGCTGCTGGCCGCTAGGGATCTGCGAT
ACCAGGGATCTATGCCCACTCTGTTAGACCTCGCTTGCGCTGTTGAGCTGACCCAT
ACCGCCTCTCTCATGCTGGACGACATGCCATGTATGGACAACGCTGAGCTGCGGCG
AGGCCAGCCTACAACACACAAGAAGTTCGGAGAGTCGGTTGCAATCCTAGCTTCG
GTCGGACTCCTTTCTAAGGCCTTCGGATTGATTGCCGCCACCGGAGATCTCCCTGG
CGAGAGAAGAGCGCAAGCCGTTAATGAGCTGTCCACCGCTGTTGGAGTCCAAGGC
TTGGTACTTGGTCAGTTCAGAGATCTGAACGACGCGGCTCTGGACCGTACACCTG

ACGCTATTCTCAGTACCAACCATCTCAAGACCGGAATCCTCTTTAGCGCCATGCTAC
AGATTGTCGCTATCGCCTCTGCCTCCTCTCCCTCCACGCGAGAAACCCTCCATGCG
TTTGCCCTTGACTTTGGTCAGGCCTTCCAGCTCCTCGACGACCTGCGGGATGATCA
CCCGGAGACTGGCAAAGATAGAAACAAGGATGCCGGTAAGTCTACCCTTGTTAAC
CGACTCGGCGCCGACGCGGCTCGACAGAAGCTGCGCGAGCACATCGACAGCGCA
GACAAGCATCTGACGTTCGCTTGTCCACAGGGAGGTGCTATTCGACAGTTCATGCA
CCTGTGGTTTGGCCACCACTTGGCTGATTGGAGCCCTGTCATGAAGATTGCCTAGT
CTATGAGACCTGAGACGgcat

GGA-IDI (*isopentenyl diphosphate isomerase - idi)*

gcatCGTCTCATCGGTCTCAAATGAAGGACGAGAGACTGGTCCAGCGAAAGAACGA
TCACCTTGATATCGTTTTGGACCCGCGCCGTGCTGTTACTCAGGCCTCCGCGGGATT
TGAGCGATGGCGTTTTACGCACTGTGCTCTCCCGGAGTTGAACTTCTCAGATATCA
CGCTTGAAACGACCTTTTTGAACCGACAGCTTCAGGCCCCGCTGCTAATTTCTTCC
ATGACTGGAGGTGTTGAGCGCTCCCGACACATCAATCGGCACCTTGCCGAGGCCG
CCCAGGTGTTGAAGATTGCCATGGGAGTCGGAAGCCAGCGAGTTGCCATCGAGAG
CGACGCCGGACTCGGCCTCGATAAGACCCTGCGGCAGCTCGCCCCAGACGTCCCA
TTGTTGGCCAACTTGGGAGCTGCCCAGCTAACTGGTCGAAAGGGAATTGATTACG
CTCGACGAGCCGTGGAAATGATTGAGGCCGACGCGCTCATCGTTCATCTGAATCCC
TTGCAGGAGGCCCTGCAGCCTGGAGGCGACAGAGACTGGCGTGGCCGACTGGCC
GCTATTGAAACACTGGTTCGTGAGCTTCCTGTCCCCCTGGTCGTTAAGGAAGTGGG
TGCTGGCATCTCTCGAACCGTGGCCGGACAGCTCATTGACGCCGGAGTCACTGTG
ATCGACGTTGCCGGAGCCGGTGGCACCTCATGGGCCGCCGTAGAGGGTGAGCGAG
CCGCCACAGAACAACAACGAAGCGTGGCTAACGTGTTCGCGGACTGGGGCATTCC
TACAGCCGAGGCTTTGGTTGACATCGCTGAGGCTTGGCCCCAGATGCCCTTGATTG
CTAGCGGTGGTATTAAGAACGGCGTGGACGCGGCAAAGGCCCTCCGACTGGGCGC
CTGCATGGTGGGACAGGCTGCTGCTGTCCTGGGCAGTGCGGGTGTGTCTACAGAG
AAAGTTATTGACCACTTTAACGTGATCATTGAGCAATTGCGAGTTGCATGCTTCTGT
ACTGGCTCCAGATCTCTTTCGGATCTTAAGCAAGCTGACATTCGATACGTTCGGGA
CACACCCTGATCTATGAGACCTGAGACGgcat

Sequences used for Gibson Assembly construction of the modified replicative vector

pHR-PS (*phytoene synthase;* WP_312633718.1*)* – TEF promoter – PS gene – XPR2 terminator

AGAGACCGGGTTGGCGGCGTATTTGTGTCCCAAAAAACAGCCCCAATTGCCCCAA
TTGACCCCAAATTGACCCAGTAGCGGGCCCAACCCCGGCGAGAGCCCCCTTCACC
CCACATATCAAACCTCCCCCGGTTCCCACACTTGCCGTTAAGGGCGTAGGGTACTG
CAGTCTGGAATCTACGCTTGTTCAGACTTTGTACTAGTTTCTTTGTCTGGCCATCCG
GGTAACCCATGCCGGACGCAAAATAGACTACTGAAAATTTTTTTGCTTTGTGGTTG
GGACTTTAGCCAAGGGTATAAAAGACCACCGTCCCCGAATTACCTTTCCTCTTCTTT

TCTCTCTCTCCTTGTCAACTCACACCCGAAATCGTTAAGCATTTCCTTCTGAGTATA
AGAATCATTCAAAATGAAGAAGACAGTCGTAATCGGCGCCGGATTCGGTGGCTTG
GCTCTTGCAATTCGACTACAGGCGGCTGGTATTCCTACCGTCCTCCTGGAGCAGCG
GGATAAGCCTGGAGGCCGAGCCTATGTGTGGCACGACCAGGGATTCACTTTCGAC
GCCGGACCCACGGTTATCACTGACCCTACTGCGCTGGAGGCTCTTTTCACACTCGC
TGGCCGGCGCATGGAAGATTACGTGAGACTGCTGCCTGTAAAGCCATTCTACCGAC
TGTGTTGGGAGTCTGGAAAGACTCTGGACTATGCCAACGATTCTGCTGAGCTTGA
AGCTCAAATCACTCAGTTTAACCCCAGGGATGTCGAGGGATACCGACGGTTCCTTG
CCTACTCTCAAGCAGTCTTCCAAGAGGGGTACCTGCGTCTGGGATCTGTGCCCTTT
CTTTCTTTTCGAGACATGCTTAGAGCTGGGCCCCAGCTCTTGAAGCTGCAGGCATG
GCAATCCGTGTACCAATCGGTCAGCCGGTTCATCGAGGATGAGCATCTCCGACAGG
CATTTTCTTTCCACTCCCTTCTGGTGGGAGGAAACCCCTTCACTACTTCCTCTATCT
ACACTCTAATCCACGCCCTGGAGCGAGAATGGGGCGTGTGGTTTCCCGAGGGAGG
TACCGGCGCGCTTGTTAACGGAATGGTGAAGCTCTTCACAGACTTGGGCGGCGAG
ATAGAGCTTAATGCGCGAGTTGAAGAGCTGGTGGTAGCAGACAATAGAGTATCCCA
GGTCCGACTGGCCGATGGCCGAATCTTCGACACGGATGCAGTGGCTTCTAACGCC
GATGTGGTCAATACATATAAGAAGCTCCTGGGACACCACCCCGTCGGACAAAAGC
GCGCCGCTGCCCTAGAGCGGAAGTCGATGTCCAACTCGCTGTTCGTTCTGTACTTT
GGACTGAACCAGCCCCATTCCCAGCTAGCTCATCACACCATCTGCTTTGGCCCGCG
GTACCGGGAGCTCATTGATGAGATCTTCACCGGGTCGGCTCTAGCCGACGATTTCT
CTTTGTACCTTCATTCTCCCTGTGTTACCGACCCTTCCCTTGCCCCTCCCGGGTGTG
CCTCGTTCTATGTGCTCGCCCCTGTGCCTCATCTGGGTAACGCGCCTCTGGATTGGG
CCCAGGAGGGTCCCAAGCTCCGTGACCGTATTTTTGACTACCTGGAGGAGCGATAT
ATGCCCGGTCTGCGTTCTCAGCTGGTCACTCAACGAATCTTTACACCGGCCGACTT
CCACGACACCCTGGACGCTCACCTTGGATCGGCTTTCTCTATTGAACCTCTCCTTAC
CCAGAGTGCCTGGTTTCGACCACATAACCGAGACTCGGATATTGCTAACCTGTATC
TCGTCGGAGCTGGCACACATCCGGGAGCCGGCATTCCGGGAGTGGTCGCTTCTGC
AAAGGCTACTGCCTCGCTGATGATCGAGGACCTGCAATGAGTGTCTGTGGTATCTA
AGCTATTTATCACTCTTTACAACTTCTACCTCAACTATCTACTTTAATAAATGAATATC
GTTTATTCTCTATGATTACTGTATATGCGTTCCTCTAAGACAAATCG

pHR-PD (*phytoene desaturase)* TEF promoter – PD gene – XPR2 terminator

AGAGACCGGGTTGGCGGCGTATTTGTGTCCCAAAAAACAGCCCCAATTGCCCCAA
TTGACCCCAAATTGACCCAGTAGCGGGCCCAACCCCGGCGAGAGCCCCCTTCACC
CCACATATCAAACCTCCCCCGGTTCCCACACTTGCCGTTAAGGGCGTAGGGTACTG
CAGTCTGGAATCTACGCTTGTTCAGACTTTGTACTAGTTTCTTTGTCTGGCCATCCG
GGTAACCCATGCCGGACGCAAAATAGACTACTGAAAATTTTTTTGCTTTGTGGTTG
GGACTTTAGCCAAGGGTATAAAAGACCACCGTCCCCGAATTACCTTTCCTCTTCTTT
TCTCTCTCTCCTTGTCAACTCACACCCGAAATCGTTAAGCATTTCCTTCTGAGTATA
AGAATCATTCAAAATGTCCCAGCCTCCCTTGCTAGACCACGCCACTCAGACCATGG
CAAACGGATCTAAATCTTTTGCCACGGCCGCTAAGTTGTTCGACCCCGCTACCAGG
AGATCGGTCCTGATGCTTTACACCTGGTGTCGACACTGTGACGACGTCATTGACGA
CCAGACCCATGGCTTCGCTTCCGAGGCCGCCGCTGAGGAGGAGGCTACCCAACGA

CTGGCCCGACTCCGTACCCTAACTCTGGCGGCTTTCGAGGGCGCGGAGATGCAGG
ACCCCGCCTTCGCGGCCTTTCAGGAAGTTGCACTTACCCACGGGATTACGCCTCGA
ATGGCTCTCGACCATCTGGATGGATTCGCCATGGACGTCGCTCAAACACGATACGT
GACATTCGAAGACACTCTACGCTACTGCTATCACGTTGCCGGTGTGGTTGGTCTGA
TGATGGCCAGAGTGATGGGTGTGCGAGACGAACGAGTGCTGGACAGAGCCTGCG
ACCTTGGACTTGCCTTCCAGCTCACGAATATCGCTCGGGACATCATTGACGACGCC
GCTATTGATCGTTGCTACCTGCCCGCTGAGTGGTTGCAAGATGCGGGCTTGACACC
CGAGAACTACGCTGCCCGAGAAAACAGAGCTGCGCTCGCTCGAGTCGCTGAGCG
GCTGATTGACGCCGCTGAACCATACTACATTTCCAGTCAGGCTGGCCTACATGATTT
GCCCCCCAGATGCGCCTGGGCCATTGCAACAGCGCGGAGTGTTTACCGAGAAATC
GGTATCAAGGTCAAAGCCGCTGGTGGATCGGCCTGGGACCGTAGACAGCACACCT
CTAAGGGCGAAAAAATCGCCATGCTGATGGCCGCGCCGGGACAGGTCATTCGGGC
GAAGACAACAAGGGTCACGCCACGACCAGCCGGGCTTTGGCAGAGACCAGTCTA
GGTGTCTGTGGTATCTAAGCTATTTATCACTCTTTACAACTTCTACCTCAACTATCTA
CTTTAATAAATGAATATCGTTTATTCTCTATGATTACTGTATATGCGTTCCTCTAAGAC
AAATCG

pHR-GGDS (*geranylgeranyl diphosphate synthase - crtE)* – TEF promoter – GGDS gene – LIP2 terminator

AGAGACCGGGTTGGCGGCGTATTTGTGTCCCAAAAAACAGCCCCAATTGCCCCAA
TTGACCCCAAATTGACCCAGTAGCGGGCCCAACCCCGGCGAGAGCCCCCTTCACC
CCACATATCAAACCTCCCCCGGTTCCCACACTTGCCGTTAAGGGCGTAGGGTACTG
CAGTCTGGAATCTACGCTTGTTCAGACTTTGTACTAGTTTCTTTGTCTGGCCATCCG
GGTAACCCATGCCGGACGCAAAATAGACTACTGAAAATTTTTTTGCTTTGTGGTTG
GGACTTTAGCCAAGGGTATAAAAGACCACCGTCCCCGAATTACCTTTCCTCTTCTTT
TCTCTCTCTCCTTGTCAACTCACACCCGAAATCGTTAAGCATTTCCTTCTGAGTATA
AGAATCATTCAAAGATGGTCTCTGGATCTAAGGCAGGGGTGAGTCCTCACCGGGA
GATTGAGGTGATGCGTCAGTCAATCGACGACCATCTGGCGGGGCTGCTGCCCGAA
ACCGACAGCCAGGACATCGTCTCCCTGGCCATGCGAGAGGGCGTGATGGCTCCCG
GGAAGCGAATTCGGCCACTTCTGATGCTGCTGGCCGCTAGGGATCTGCGATACCAG
GGATCTATGCCCACTCTGTTAGACCTCGCTTGCGCTGTTGAGCTGACCCATACCGC
CTCTCTCATGCTGGACGACATGCCATGTATGGACAACGCTGAGCTGCGGCGAGGCC
AGCCTACAACACACAAGAAGTTCGGAGAGTCGGTTGCAATCCTAGCTTCGGTCGG
ACTCCTTTCTAAGGCCTTCGGATTGATTGCCGCCACCGGTGACCTCCCTGGCGAGA
GAAGAGCGCAAGCCGTTAATGAGCTGTCCACCGCTGTTGGAGTCCAAGGCTTGGT
ACTTGGTCAGTTCAGAGATCTGAACGACGCGGCTCTGGACCGTACACCTGACGCT
ATTCTCAGTACCAACCATCTCAAGACCGGAATCCTCTTTAGCGCCATGCTACAGATT
GTCGCTATCGCCTCTGCCTCCTCTCCCTCCACGCGAGAGACCCTCCATGCGTTTGC
CCTTGACTTTGGTCAGGCCTTCCAGCTCCTCGACGACCTGCGGGATGATCACCCGG
AGACTGGCAAAGATAGAAACAAGGATGCCGGTAAGTCTACCCTTGTTAACCGACT
CGGCGCCGACGCGGCTCGACAGAAGCTGCGCGAGCACATCGACAGCGCAGACAA
GCATCTGACGTTCGCTTGTCCACAGGGAGGTGCTATTCGACAGTTCATGCACCTGT
GGTTTGGCCACCACTTGGCTGATTGGAGCCCTGTCATGAAGATTGCCTGAGTGTCT

GTGGTATCTAAGCTATTTATCACTCTTTACAACTTCTACCTCAACTATCTACTTTAAT
AAATGAATATCGTTTATTCTCTATGATTACTGTATATGCGTTCCTCTAAGACAAATCG

pHR-IDI (*isopentenyl diphosphate isomerase - idi*) – TEF promoter – IDI gene – LIP2 terminator

AGAGACCGGGTTGGCGGCGTATTTGTGTCCCAAAAAACAGCCCCAATTGCCCCAA
TTGACCCCAAATTGACCCAGTAGCGGGCCCAACCCCGGCGAGAGCCCCCTTCACC
CCACATATCAAACCTCCCCCGGTTCCCACACTTGCCGTTAAGGGCGTAGGGTACTG
CAGTCTGGAATCTACGCTTGTTCAGACTTTGTACTAGTTTCTTTGTCTGGCCATCCG
GGTAACCCATGCCGGACGCAAAATAGACTACTGAAAATTTTTTTGCTTTGTGGTTG
GGACTTTAGCCAAGGGTATAAAAGACCACCGTCCCCGAATTACCTTTCCTCTTCTTT
TCTCTCTCTCCTTGTCAACTCACACCCGAAATCGTTAAGCATTTCCTTCTGAGTATA
AGAATCATTCAAAATGAAGGACGAGAGACTGGTCCAGCGAAAGAACGATCACCTT
GATATCGTTTTGGACCCGCGCCGTGCTGTTACTCAGGCCTCCGCGGGATTTGAGCG
ATGGCGTTTTACGCACTGTGCTCTCCCGGAGTTGAACTTCTCAGATATCACGCTTG
AAACGACCTTTTTGAACCGACAGCTTCAGGCCCCGCTGCTAATTTCTTCCATGACT
GGAGGTGTTGAGCGCTCCCGACACATCAATCGGCACCTTGCCGAGGCCGCCCAGG
TGTTGAAGATTGCCATGGGAGTCGGAAGCCAGCGAGTTGCCATCGAGAGCGACGC
CGGTCTCGGCCTCGATAAGACCCTGCGGCAGCTCGCCCCAGACGTCCCATTGTTGG
CCAACTTGGGAGCTGCCCAGCTAACTGGTCGAAAGGGAATTGATTACGCTCGACG
AGCCGTGGAAATGATTGAGGCCGACGCGCTCATCGTTCATCTGAATCCCTTGCAGG
AGGCCCTGCAGCCTGGAGGCGACAGAGACTGGCGTGGCCGACTGGCCGCTATTGA
AACACTGGTTCGTGAGCTTCCTGTCCCCCTGGTCGTTAAGGAAGTGGGTGCTGGC
ATCTCTCGAACCGTGGCCGGACAGCTCATTGACGCCGGCGTCACTGTGATCGACGT
TGCCGGAGCCGGTGGCACCTCATGGGCCGCCGTAGAGGGTGAGCGAGCCGCCAC
AGAACAACAACGAAGCGTGGCTAACGTGTTCGCGGACTGGGGCATTCCTACAGCC
GAGGCTTTGGTTGACATCGCTGAGGCTTGGCCCCAGATGCCCTTGATTGCTAGCGG
TGGTATTAAGAACGGCGTCGACGCGGCAAAGGCCCTCCGACTGGGCGCCTGCATG
GTGGGACAGGCTGCTGCTGTCCTGGGCAGTGCGGGTGTGTCTACAGAGAAAGTTA
TTGACCACTTTAACGTGATCATTGAGCAATTGCGAGTTGCATGCTTCTGTACTGGCT
CCAGATCTCTTTCGGATCTTAAGCAAGCTGACATTCGATACGTTCGGGACACACCC
TGAGTGTCTGTGGTATCTAAGCTATTTATCACTCTTTACAACTTCTACCTCAACTATC
TACTTTAATAAATGAATATCGTTTATTCTCTATGATTACTGTATATGCGTTCCTCTAAG
ACAAATCG

2. Primers used in this experiment

The primers listed in Table S1 were used to amplify the genes for construction of JMP62 type vectors with single genes overexpression as well as for transformants verification and RT-qPCR.

Table S1. List of primers used in this study.

Name of primer	Sequence
PD-BamHI-F	atcgGGATCCATGAAGAAAACAGTCGTAATCGGCG
PD-AvrII-R	gctaCCTAGGTCATTGCAGGTCCTCGATCATCAG
PS-BamHI-F	gatcGGATCCATGTCCCAGCCTCCCTTGCTAG
PS-AvrII-R	gatcCCTAGGTTAGACTGGTCGCTGCCAAAGC
GGDS-BamHI-F	gctaGGATCCATGGTGTCTGGATCTAAGGCAGGG
GGDS-AvrII-R	tagcCCTAGGCTAGGCAATCTTCATGACAGGGC
IDI-BamHI-F	actgGGATCCATGAAGGACGAGAGACTGGTCCAG
IDI-AvrII-R	tagcCCTAGGTCAGGGTGTGTCCCGAACG
TEF-F	CTCCTTGTCAACTCACACCCG
LIP2-R	CTTAGAGGAACGCATATACAGTAATCATAGAG
**RT-qPCR**	
crtI-rev	AACACTGCTTGAGAGTAGGCAA
crtI-fwd	CTGTGTTGGGAGTCTGGAAAGA
crtB-rev	CAGGTAGCAACGATCAATAGCG
crtB-fwd	GTCTGATGATGGCCAGAGTGAT
crtE-rev	TTCTATCTTTGCCAGTCTCCGG
crtE-fwd	CTACAGATTGTCGCTATCGCCT
IDI-rev	CTCCCATGGCAATCTTCAACAC
IDI-fwd	AACGACCTTTTTGAACCGACAG
Act-fwd	CTGGGATGACATGGAGAAGATCTGGC
Act-rev	GAGGCGTACAGGGAGAGGACG

3. Electron microscopy picture of *Y. lipolytica* wild type (W29) and phospholipid overproducing (PS05) strains growing in YNB glucose media.

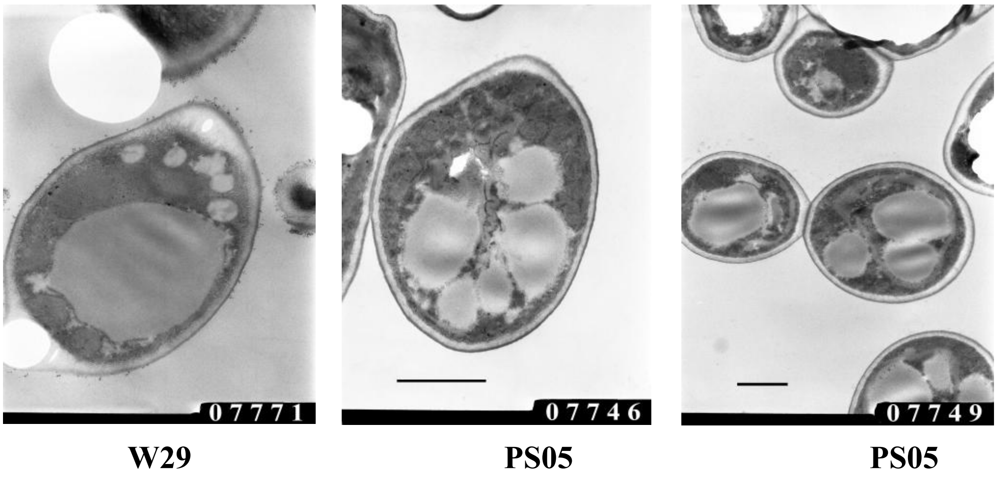

Figure S1. Morphology of *Y. lipolytica* strains (W29 – wild type, PS05 – phospholipid overproducing strain) growing in a YNB medium with glucose.

4. Carbon source-dependent colour differences in *Y. lipolytica* lycopene cultures depending on strain genotype and pathway integration method

GLUCOSE

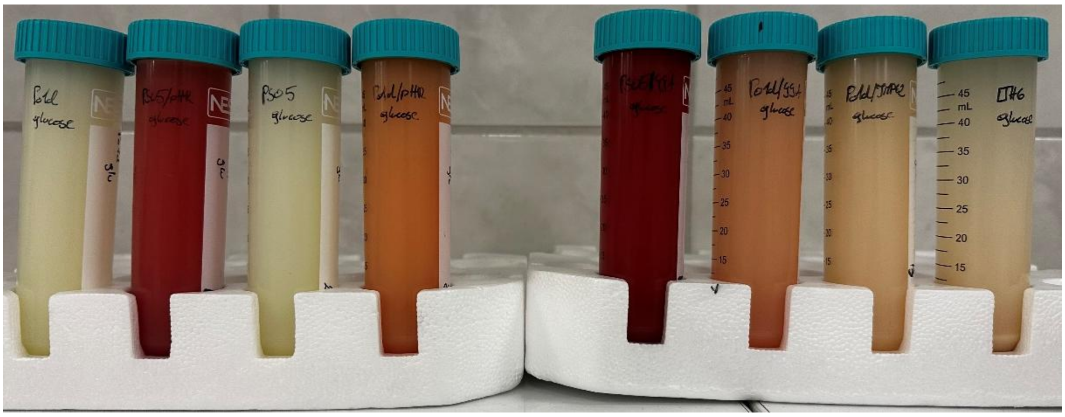

GLYCEROL

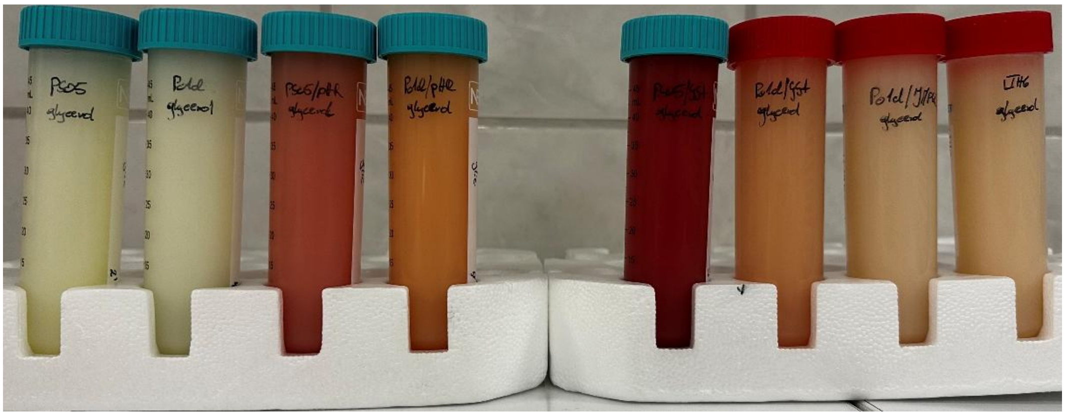

BUTYRATE

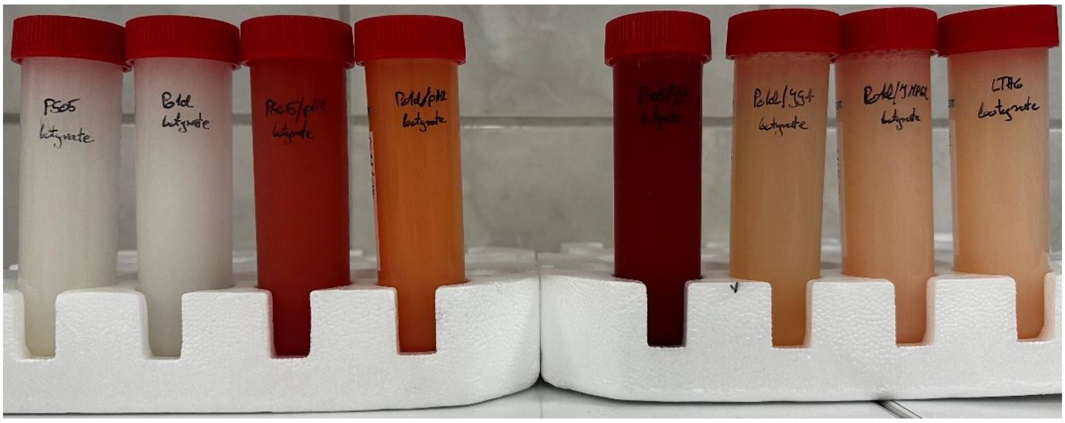

ACETATE

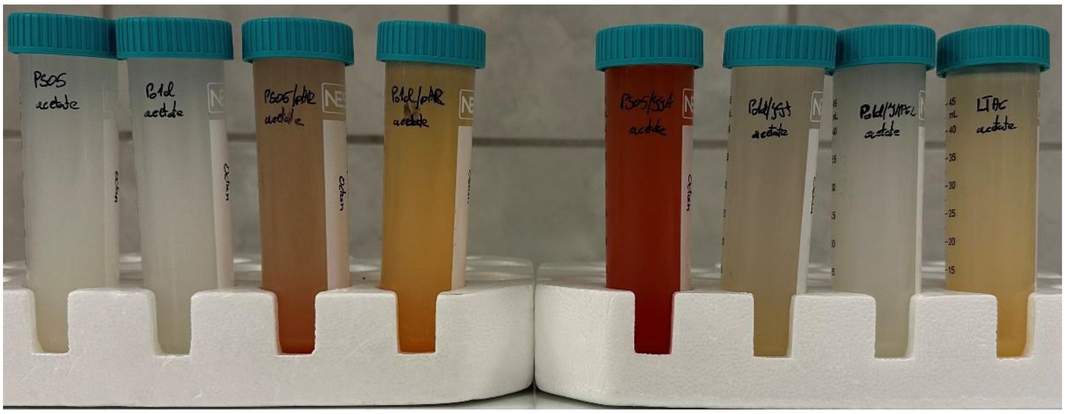

PROPIONATE

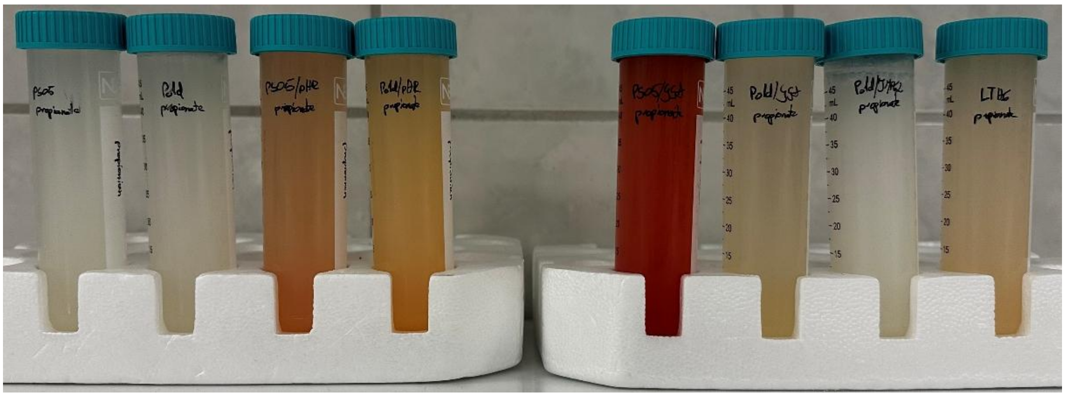

MIX (SCFA)

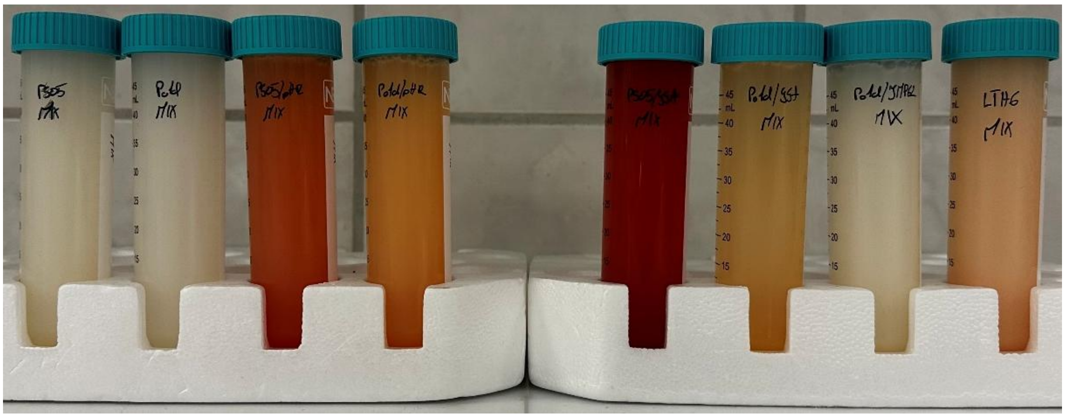

Figure S2. Effect of carbon source and genetic constructs on pigmentation and it's intensity in lycopene-producing *Y. lipolytica* transformants (order: PS05, PO1d, PS05/4lyc/pHR, PO1d/4lyc/pHR, PS05/4lyc/GGA, PO1d/4lyc/GGA, PO1d/JMP62, LTH6).

5. **Representative chromatogram of lycopene extraction from *Y. lipolytica* transformants, exemplified by strain PS05/4lyc/GGA.**

A)

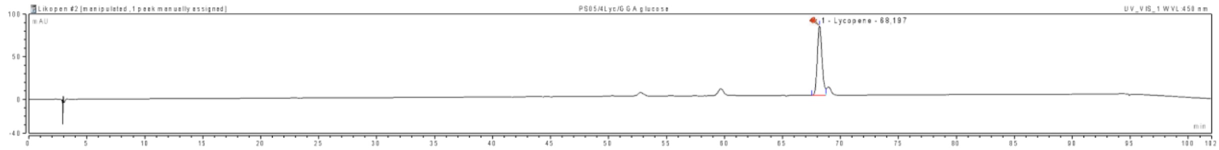

B)

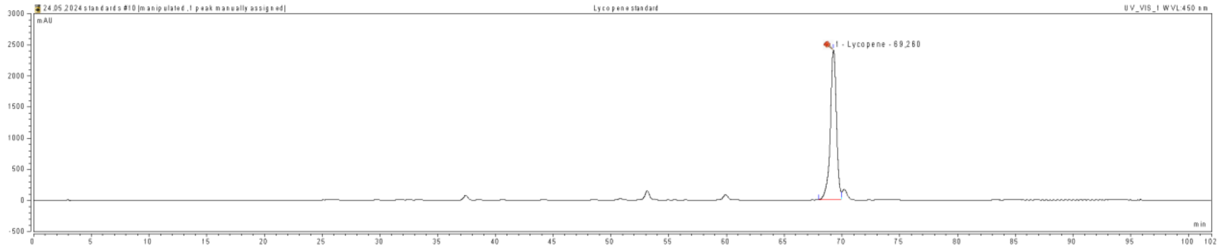

Figure S3. HPLC chromatographic profiles of lycopene. (A) Extract from *Y. lipolytica* strain PS05/4lyc/GGA. (B) Reference chromatogram of lycopene standard used for identification.
